# Advances in Research on Immunoregulation of Macrophages by Plant Polysaccharides

**DOI:** 10.3389/fimmu.2019.00145

**Published:** 2019-02-05

**Authors:** Miao Yin, Ying Zhang, Hua Li

**Affiliations:** Shandong Provincial Key Laboratory of Animal Resistance Biology, College of Life Sciences, Shandong Normal University, Jinan, China

**Keywords:** polysaccharide, immunoregulation, macrophage, receptors, plant

## Abstract

Polysaccharides are among the most important members of the biopolymer family. They are natural macromolecules composed of monosaccharides. To date, more than 300 kinds of natural polysaccharide compounds have been identified. They are present in plants, animals, and microorganisms, and they engage in a variety of physiological functions. In the 1950s, due to the discovery of their immunoregulatory and anti-tumor activities, polysaccharides became a popular topic of research in pharmacology, especially in immunopharmacology. Plants are an important source of natural polysaccharides. Pharmacological and clinical studies have shown that plant polysaccharides have many functions, such as immune regulation, anti-tumor activity, anti-inflammatory activity, anti-viral functions, anti-radiation functions, and a hypoglycaemic effect. The immunomodulatory effects of plant polysaccharides have received much attention. Polysaccharides with these effects are also referred to as biological response modifiers (BRMs), and research on them is one of the most active areas of polysaccharide research. Thus, we summarize immunomodulatory effects of botanical polysaccharides isolated from different species of plants on the macrophage. The primary effect of botanical polysaccharides is to enhance and/or activate macrophage immune responses, including increasing reactive oxygen species (ROS) production, and enhancing secretion of cytokines and chemokines. Therefore, it is believed that botanical polysaccharides have significant therapeutic potential, and represent a new method for discovery and development of novel immunomodulatory medicine.

## Introduction

A variety of polysaccharides have been discovered in different species of plants, and information about the structures and functional activities of some polysaccharides has been elucidated. It was found that the vast majority of plant polysaccharides are relatively non-toxic and do not cause significant side effects. Side effects are indeed an unsolved problem for the clinical application of immunomodulating polysaccharides derived from microorganisms and chemical synthesis. Plant polysaccharides thus represent an ideal alternative for immune modulation.

Most recent studies of plant polysaccharides have focused on separation and purification, analysis of the monosaccharide composition, structural analysis (including primary and advanced structures), and the relationship between structure and function (1–5). Table 1 shows the structures and biological activities of some plant polysaccharides. The range of the molecular weights of the polysaccharides is relatively large. The molecular weight of polysaccharide PAC-I is 10,000 kDa (6), and the molecular weight of FPS-1 is 14 kDa (8). Some polysaccharides are composed of complex sets of monosaccharides, whereas others have only one monosaccharide component. For example, *Cistanche deserticola* is made of glucose (9), and the monosaccharide component of PSPP is dextran (12), whereas CPE is made of arabinose, galactose, glucose, mannose, rhamnose, and xylose (10). The chemical structures and physiological activities of polysaccharides are related. Most polysaccharides with aβ-helix structure have strong biological functions. The glycosidic bonds of plant polysaccharides are mainly α-(1 → 6)-D, α-(1 → 4)-D, and β-(1 → 4)-D. It has been found that even the polysaccharides derived from the same plant may be of different types. For example, the polysaccharides PAC-I, PAC-II, and PAC-III, which have different molecular weights, are extracted from *Aloe vera* L., and their monosaccharide compositions also differ (24).

**Table 1:** The information of botanical polysaccharides.

**Botany**	**Polysaccharides**	**Molecular weight**	**Ingredient**	**Glucosidic bond**	**Activity**
*Aloe vera* L. (6)	PAC-I	10,000 kDa	90% Mannose, galactose, glucose, arabinose	β-(1 → 4)-D- mannose	Anti-tumor, stimulate T and B cell proliferation, promote the production of IL-1β, TNF-α, IL-2, IL-6, and IFN-γ
	PAC-II	1,300 kDa	90% Mannose, galactose, glucose arabinose	β-(1 → 4)-D- mannose	
	PAC-III	470 kDa	54% Mannose, 33% galactose, 13% arabinose		
*Angelica sinensis* (7)	ASP1		Glucose, galactose, arabinose		Radioprotection effect (ASP3)
	ASP2 ASP3	34 kDa (ASP3)	Galacturonic acid, rhamnose, galactose, arabinose		
*Aconitum carmichaeli* (8)	FPS-1	14 kDa		α-(1 → 6)-D- glucan	Stimulate lymphocyte proliferation in mice
*Cistanche deserticola* (9)			Glucose	α-(1 → 6)-D- glucan	
*Curcuma xanthorrhiza* (10)	CPE	33 kDa	18.69% Arabinose, 14% galactose, 50.67% glucose, 12.97% mannose, 2.73% rhamnose, 0.94% xylose		Activate macrophages
*Ixeris polycephala* (11)	KMCP	1,950 kDa	Arabinogalactan		Stimulate the synthesis of NO in macrophage
*Ipomoea batatas* (12)	PSPP	53.2 kDa	Glucan	α-(1 → 6)-D- glucan	Stimulates lymphocyte proliferation, activate macrophages and NKcells
*Juniperusscopolorum* (13)	J-I- J-V	30–680 kDa	Arabogalactan		J-I, J-II, J-III activate macrophages
*Phyllanthusniruri* (14)			Rhamnose, arabinose, xylose, galactose, glucose	α-(1 → 4)-D- glucan	Stimulates the production of superoxide enzymes
*Cucurbita moschata* (15)			Glucose, glucuronic acid, galactose arabinose		Antioxidant activity
*Spondias cytherea* (16)	sFTS		Rhamnose, arabinose, galactose, galacturonic acid		Activate macrophages
*Vernonia kotschyana* (17, 18)	Vk100A2a	20 kDa	Rhamnose, arabinose, galactose		Complement binding activity, promote B cell proliferation
	Vk100A2b	115 kDa			Induce chemotaxis of macrophages, Nk cells and Tcells
*Panax notoginseng* (19)		1,140 kDa	Polygalacturonic acid, rhamnose, galactan		Complement binding activity
*Rosa rugosa* (20)	P1-a	150 kDa			Inhibition reverse transcriptase activity of HIV-1
*Salvia miltiorrhiza* (21)	SMP 1	1,390 kDa		α-(1 → 6)-D- glucan	
	SMP 0.5	403 kDa		α-(1 → 6)-D- glucan	
*Tinospora cordifolia* (22)	RR1		Glucan	α-(1 → 4)-D- glucan	Activate macrophages
*Thamnolia vermicularis* (23)	Ths-2 Ths-4, Ths-5 thamnolan		Glucan Furan galactose Furan galactose		Ths-2, Ths-5, and thamnolan stimulate the proliferation of mouse spleen cells; Ths-4 and Ths-5 activate mouse peritoneal macrophages; Ths-2, Ths-4, and Ths-5 stimulated IL-10 release in spleen cells of mice

The activities of plant polysaccharides include immune regulation, anti-tumor, anti-radiation, and anti-viral effects (21, 25–27), and the structures of polysaccharides are directly related to their functional activities (28). Kralovec et al. reported that a polysaccharide/glycoprotein complex with a molecular weight >100 kDa and consisting mainly of galactose, rhamnose, and arabinose has high biological activity (29). Lo et al. proposed that arabinose, mannose, xylose, and galactose are the four most important monosaccharide components contributing to macrophage stimulating activity, whereas glucose, as the most common monosaccharide component, showed no clear role in the immunoactivity of polysaccharides (30).

## Immunomodulatory Effects of Plant Polysaccharides

Numerous studies have shown that plant polysaccharides can regulate the immune system in multiple ways and at multiple levels. They not only activate immune cells, including T cells, B lymphocytes, macrophages, and natural killer cells, but they also activate complements and promote the production of cytokines, thus showing regulatory effects on the immune system in multiple ways (25, 31–34). Innate immune regulation has an important impact on the host's ability to respond rapidly to pathogens. As important members of the immune defense system of the host, macrophages can collaborate with other types of cells (such as neutrophils) to resist external adverse factors (35–40). The immunomodulatory effects of plant polysaccharides on macrophages are mainly achieved through the generation of reactive oxygen species (ROS), the secretion of cytokines, cell proliferation, and the phagocytic activity of macrophages (38).

### Effect of Plant Polysaccharides on NO Production by Macrophages

NO is an important biologically active substance. In addition to being an important signal transducer in the central nervous system, it is widely involved in the physiological and pathological processes of multiple systems, including immune responses, and inflammatory reactions (26, 32, 41–43). In the immune system, when macrophages are stimulated and activated, a large amount of nitric oxide (NO) is released, which can kill microorganisms, parasites, and tumor cells; it can also induce inflammatory reactions and protect the body from external adverse factors (38).

Park reported that the acidic polysaccharide component BRP-4, extracted from *Basella rebra* L., can promote NO production in macrophages RAW264.7 at a dose of 10–100 μg/ml (44). A study by Luo et al. also showed that KMCP, a polysaccharide component extracted from *Ixeris polycephala*, can enhance macrophage-mediated non-specific immune responses by increasing NO production in macrophages (11). Polysaccharides extracted from *Pterospartum tridentatum* (L.) Willk. can also promote NO production in an *in vitro* culture of macrophages (45).

Similarly, the polysaccharides ASP (46), KMCP (11), SF1, SF2 (47), SPS (48), SBF (49), PG (50), and SHE (51) can stimulate NO release in mouse peritoneal macrophages and/or macrophage RAW264.7, in which SBF inhibits the release of NO in LPS-activated macrophages as well as the release of cytokines and activity mediators (49). Polysaccharides GG, SGG, and BPs inhibit the release of NO in LPS-activated macrophages RAW264.7, which suggests that these polysaccharides have similar immunomodulatory activity (36, 45).

### Effect of Plant Polysaccharides on Cytokine Secretion by Macrophages

Cytokines play an important role in the regulation of cell-cell interactions, cell growth, and cell differentiation. Cytokines can be classified based on function as interleukins (IL), tumor necrosis factors (TNF), interferons (IFN), and colony stimulating factors (CSF). They play important roles in the regulation of inflammatory responses and immune responses, and they have regulatory effects on both innate immunity and adaptive immunity (36).

The *Citrus unshiu* polysaccharide component CPE-II can significantly increase the production of the pro-inflammatory cytokines TNF-α and IL-6 and the anti-inflammatory cytokine IL-12 in macrophage RAW264.7 (52). Thus, plant polysaccharides can simultaneously regulate the expression of pro- and anti-inflammatory cytokines (IL-12 acts as a negative feedback factor to prevent the over-activation of macrophages in the hyper-inflammatory response), which suggests that the body has a self-regulatory mechanism to maintain an equilibrium state (53). Kouakou et al. found that the polysaccharide component AP-AU-1, which is extracted from *Alchornea cordifolia*, downregulates the expression of cytokines such as IL-1β, IL-6, IL-10, TNF-α, and GM-CSF in *in vitro* cultures of human and mouse macrophages (25). When LPS-activated macrophages were treated with BPs, the release of cytokines in the macrophages was inhibited, and the inhibition rates of TNF-α, IL-1β, and IL-6 were 50, 89, and 97, respectively (36). Similarly, polysaccharides such as DIP and MSE can promote the release of cytokines in macrophages (26, 54).

The synergistic effect of cytokines and plant polysaccharides on macrophages can also activate the immune response of macrophages. Im et al. found that the synergistic effect of polysaccharides SHP and IFN-γ could stimulate macrophages to release a large number of cytokines such as TNF-α, IL-1β, and NO. The transcript levels of cytokines TNF-α and IL-1 were also significantly increased. In addition, the synergistic effect caused changes in the differentiation antigens CD11b, CD18, and CD24, which are expressed by macrophages (55).

## Mechanism of Immunoregulation by Plant Polysaccharides

**Figure 1:**
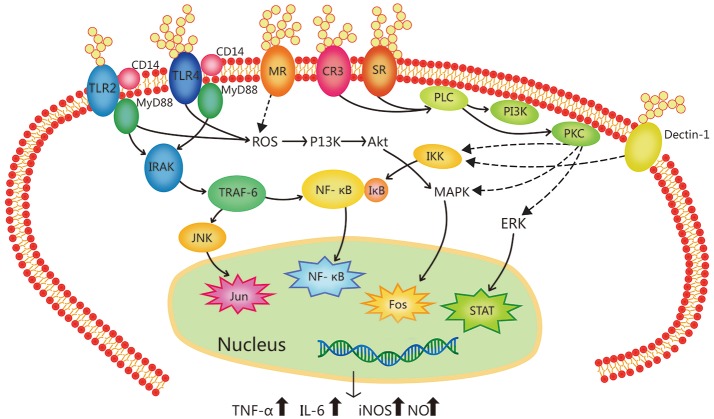
Signal transduction pathway related to the immune-regulation of botanical polysaccharides in macrophage activation. Botanical polysaccharides can activate macrophages via different kinks of receptors, such as Toll-like receptor 4 (TLR4), Toll-like receptor 2 (TLR2), complement receptor 3 (CR3), mannose receptor (MR), scavenger receptor (SR), and Dectin-1. These receptors can work separately, and some different receptor types can cooperate with each other forming clusters of signaling complexes. (e.g., TLR4-CD14, TLR2-CD14, etc.) TLR4 and TLR2 ligation leads to the activation of IL-1R associated kinase (IRAK) via an adaptor myeloid differentiation protein 88 (MyD88), with subsequent activation of TNF receptor-associated factor 6 (TRAF-6), MAP kinases (e.g., p38 and JNK) and NF-κB. It can also activating phosphoinositide-3-kinase (PI3K)-Akt pathway via reactive oxygen species (ROS), then leading to activation of the mitogen-activated protein kinase (MAPK). SR- and CR3-activated signaling pathways lead to phospholipase C (PLC) activating, whose products activate protein kinase C (PKC) and PI3K, leading to activation of the MAPK, extracellular signal regulated kinase (ERK). Activated PKC can made IKK to be phosphorylated, IKK complex phosphorylates I-κB which leading the I-κB uniquitylated and degradated. In the end, nuclear factor-κB (NF-κB) liberated. Ultimately, these activating factors enter the nucleus cause induction of gene transcription. Activation of these transcription pathways induce expression of pro-inflammatory cytokines (TNF-α, IL-6, etc.) and inducible nitric oxide synthase (iNOS).

The study of mechanisms of immunoregulation by plant polysaccharides has reached the molecule and receptor level. Most research has shown that plant polysaccharides activate macrophages by recognizing and binding to specific receptors on the surfaces of macrophages, which initiates the immune response and exerts an immunomodulatory effect. These macrophage receptors are called pattern recognition molecules. Macrophages can bind to plant-derived polysaccharides and/or glycoproteins by Toll-like receptor 4 (TLR4), CD14, complement receptor 3 (CR3), scavenger receptor (SR), mannose receptor (MR), and Dectin-1. The activation of macrophage receptors can initiate a series of intracellular signaling cascades, leading to the transcriptional activation and production of inflammation-related cytokines (56–60), as shown in the Figure 1 (35).

### TLR4 Mediates the Activation of Macrophages by Plant Polysaccharides

TLR4 is a very important class of membrane receptors that are expressed on the surfaces of macrophages and mediate the activation of macrophages by converting extracellular signals (61–64). The binding of TLR4 to the ligand results in the formation of a complex between the TLR cytoplasmic domain, the adaptor protein myeloid differentiation primary response gene 88 (MyD88), and the interleukin-1 receptor-associated kinase (IRAK), which in turn activates tumor necrosis factor receptor-associated factor 6 (TRAF6) and finally initiates the mitogen-activated protein kinase (MAPK) signaling pathway (65–67). Polysaccharide CPE-II acts on RAW264.7 via TLR4 and TLR2, and it influences the level of IL-6 and NO by regulating MAPKs and nuclear factor-κB (NF-κB) (52). Similarly, the effect of G1-4A on macrophage RAW264.7 is related to TLR4. An experiment showed that macrophage activity could be decreased after blocking TLR4 activity using siRNA and antibodies, which suggests that G1-4A activates macrophages via a TLR4-MyD88-dependent pathway (22, 68).

Studies have shown that polysaccharide PG can activate macrophages through TLR4 (37, 69). Because activator protein 1 (AP-1) and MAPK are important downstream signaling molecules that mediate the activation of macrophages and the expression of inflammatory genes, T. Yeo Dae Yoon et al. investigated the effect of PG on AP-1 and MAPK activity in the macrophage RAW264.7. The electrophoretic mobility shift assay (EMSA) results showed that PG significantly increases the DNA binding activity of AP-1, and further experiments demonstrated that PG activates three subgroups of MAPK (ERK1/2, SAPK/JNK, and p38 MAPK) (37, 66). When the induction effect of PG was detected after pretreatment of macrophages with specific inhibitors of these three kinases, PG did not show an activation effect, and the DNA binding activity of AP-1 was inhibited by the MAPK inhibitors. This indicates that PG activates macrophages through the TLR4 signaling pathway and that MAPK and AP-1 are involved. Wang's research showed that HRWP-A could recover the body condition and activated macrophage in Cyclophosphamide (CTX)-induced immunosuppressed mice which may depended on mouse peritoneal macrophages. qPCR and western blot revealed that HRWP-A upregulated the expression of TLR4 mRNA *in vitro*, and that process was accompanied by a clear increase in MyD88 expression and p-IκB-α, but these effects were largely abrogated by pretreatment with anti-TLR4 antibodies(37). HRWP-A failed to induce the production of NO, IL-1β, and IL-6 in peritoneal macrophages prepared from C3H/HeJ mice which have a point mutation in the Tlr4 gene, suggesting the involvement of the TLR4 molecule in HRWP-A-mediated macrophage activation (70).

### CD14 and CR3 Mediate the Activation of Macrophages by Plant Polysaccharides

Studies have shown that many different receptors work together through pairing and cooperation. For example, TLR4 and CD14, Dectin-1 and TLR2, and CD14 and CR3 form complexes for signal transduction (71). CD14 is known to be a high-affinity receptor for LPS-activated macrophages. CR3 is a receptor for complement protein and β-glucan. Both are involved in the immune response to plant polysaccharides. CD14 antibody and CR3 antibody significantly reduced the amounts of NO released in PG-induced macrophages, suggesting that the surfaces of these two types of molecules may have binding sites for plant polysaccharides (72). Plant polysaccharides also affect the expression of receptors on the macrophage surface. For example, *Panax ginseng* polysaccharide significantly upregulates the expression of CD14 in mouse peritoneal macrophages and downregulates the expression of CR3 at the same time (73).

### SR-Mediated Activation of Macrophages by Plant Polysaccharides

Scavenger receptors have multiple ligands, so these receptors play important roles in pathogen clearance, host defense, and the signal transduction of macrophages (74). Studies have shown that SR and CR3 activate phospholipase C (PLC) after binding to their ligands and that the product of PLC enzymolysis activates protein kinase C (PKC) and phosphatidylinositol 3-kinase (PI3K), resulting in the activation of MAPK, extracellular signal-regulated kinase (ERK), and NF-κB, eventually triggering gene transcription events (75). PKC is a protein kinase that is commonly expressed in mammals. PKC signaling and MAPK signaling play important roles in the innate immune response of monocyte-macrophages (76).

Nakamura et al. found that the polysaccharide fucoidan, which has anticoagulant and anticancer activity, activates macrophages through SR to release NO (77). In the peritoneal macrophages of wild-type mice, fucoidan stimulates the release of NO in a dose-dependent manner, but it does not induce the production of NO in the peritoneal macrophages of SR knockout mice (SR^−/−^), indicating that fucoidan activates macrophages via the SR pathway (74, 78). Furthermore, inhibition experiments using different signal molecules showed that fucoidan increases iNOS activity in macrophages through p38 MAPK signaling and NF-κB signaling, thereby increasing the release of NO. p38 MAPK can activate multiple transcription factors, including cAMP-response element binding protein (CREB) and AP-1, and it participates in multiple processes of post-transcriptional gene regulation. Therefore, fucoidan and NF-κB may have a synergistic effect on the activation of macrophages by the p38 MAPK pathway (79).

### MR Mediates the Activation of Macrophages by Plant Polysaccharides

The mannose receptor is an important member of the C-type lectin-like receptor family. It can recognize glycosylated molecules that contain a mannose residue, a trehalose group, or an N-acetyl glucose residue (80). Many mannose receptors are expressed on the surfaces of alveolar macrophages, peritoneal macrophages, and mononuclear macrophages in blood, and they play an important role in the early immune response. After binding to plant polysaccharide ligands, mannose receptors can increase the phagocytic activity of macrophages, produce ROS, activate the transcription factor NF-κB, and induce the secretion of cytokines (81).

### Dectin-1 Mediates the Activation of Macrophages by Plant Polysaccharides

Dectin-1 is a surface receptor that is expressed in immune cells to defend against fungal pathogens. It is a type II membrane protein with a molecular weight of 28 kDa. Sequencing analysis showed that the extracellular domain of Dectin-1 is a C-type lectin-like receptor that binds β-glucan (82, 83). Dectin-1 recognizes glucans linked by β-1,3 and β-1,6 glycosidic bonds, which are derived from yeast, other fungi, plants, and bacteria, and it mediates phagocytosis and the immune response of macrophages to glucan while promoting the release of inflammatory mediators in macrophages (82, 83). Dectin-1 can also form a signaling complex with TLR2 to transmit the stimulatory signals of macrophages. During the signal transduction, the expression of Dectin-1 in the activated macrophages is significantly increased compared with that found in cells in a resting state (83).

### Other Pathways to Activate Macrophages by Plant Polysaccharides

In addition to interacting with receptors to activate macrophages, plant polysaccharides can reach the interior of macrophages via phagocytosis. Unlike starch and glycogen, the phagocytosed plant polysaccharide molecules are not easily digested completely; therefore, the incompletely degraded polysaccharides can activate macrophages as a co-stimulatory signal (84).

Although multiple signaling pathways are involved in the activation of macrophages, almost all such signals are ultimately attributed to the expression of inflammation-related cytokines and inducible nitric oxide synthase (iNOS). NF-κB is a group of pleiotropic transcription factors that are known to be activated by more than 400 stimuli, and the number of stimuli is still increasing. NF-κB is an important transcription factor regulating the immune response and inflammatory response. It mainly plays a role in regulating cell proliferation and apoptosis and immune and inflammatory responses, and it regulates the transcription of a variety of pro-inflammatory genes. iNOS is an important inflammatory gene regulated by NF-κB. Studies have shown that a variety of polysaccharides, such as APS, AP-AU, CPE, and KMCP, can enhance the transcriptional activity of iNOS by activating NF-κB, thus promoting the generation of NO in macrophages (11, 25, 52, 85).

## Perspective

The developments in mass spectrometry, GC-MS, X-ray fiber diffraction, nuclear magnetic resonance, electron diffraction, and other analytical techniques have made it possible to obtain structural information about plant polysaccharides and their mechanisms. However, there are still many unresolved issues. First, the relationship between the structure and function of plant polysaccharides is still not fully understood. The activities of polysaccharides originating from the same plant are also very different, suggesting that the structures of polysaccharides are directly related to the biological activities (59). However, due to the complex structure and wide variety of polysaccharides, the isolation, purification, and structure determination of polysaccharides are very difficult. Therefore, the structural characteristics of most of the reported active polysaccharides remain unclear.

Second, a large number of inflammatory mediators released in macrophages after activation by plant polysaccharides may cause excessive inflammation in the body. The appropriate amount of NO released by macrophages can exert beneficial functions and protect the body from adverse factors. If the macrophages receive continuous stimulation and release excessive NO, this may lead to sepsis and local or systemic inflammatory disorders. In particular, excessive inflammation is seriously harmful to human health. A large amount of experimental and clinical evidence indicates that long-term inflammation can increase the risk of cancer because a variety of inflammatory mediators can directly regulate the expression of oncogenes. In addition, key inflammation-related enzymes such as iNOS and COX-2 are associated with cancer induced by long-term inflammation (86, 87). Therefore, it is important to continue studying whether the pro- and anti-inflammatory factors induced by plant polysaccharides can maintain equilibrium in the body without causing harm due to excessive activation. The impact of this factor should be considered in clinical applications.

Third, the problem of endotoxin contamination in active plant polysaccharides needs to be excluded. Because polymyxin B can inhibit the activity of LPS, it is often used to indirectly detect the contamination of LPS. However, studies have shown that polymyxin B has different levels of impact on iNOS expression and cytokine production in addition to inhibiting LPS activity (88). Therefore, this detection method is also controversial. Finding safer and more effective methods for the detection of endotoxin contamination is another urgent problem to be solved.

Fourth, plant polysaccharides and microorganism-derived polysaccharides can bind to the same receptors on the surfaces of macrophages and induce similar immune responses, suggesting that polysaccharides from different sources may be highly conserved throughout evolutionary history. Therefore, we speculate that the response of macrophages to plant polysaccharides may mimic their natural immune responses to microbial pathogens. Although plant polysaccharides can bind to different receptors on the surfaces of macrophages, they activate similar signaling pathways. Does this mean that there is coordination or crosstalk between the signals transmitted by different receptors? These problems need to be further investigated.

With the rapid development of biology and medicine, the study of plant polysaccharides is receiving more extensive attention. Although there are still many unsolved puzzles in the field, the clinical value and broad application prospects of plant polysaccharides make them an important direction for the development of new drugs. A variety of plant polysaccharides have been used as drugs or as adjuvants to enhance drug efficacy or reduce side effects. As research progresses, the immunomodulatory mechanisms of active plant polysaccharides will be explained more clearly, and more active plant polysaccharides will be developed and utilized.

## Author Contributions

All authors listed have made a substantial, direct and intellectual contribution to the work, and approved it for publication. MY and HL wrote the manuscript together. YZ edited the manuscript.

### Conflict of Interest Statement

The authors declare that the research was conducted in the absence of any commercial or financial relationships that could be construed as a potential conflict of interest.
